# Virtual View Generation Based on 3D-Dense-Attentive GAN Networks

**DOI:** 10.3390/s19020344

**Published:** 2019-01-16

**Authors:** Junwei Fu, Jun Liang

**Affiliations:** State Key Lab of Industrial Control Technology, Zhejiang University, Hangzhou 310027, China; fujunwei@zju.edu.cn

**Keywords:** image regeneration, stereo image processing, generative adversarial networks, vehicle safety

## Abstract

A binocular vision system is a common perception component of an intelligent vehicle. Benefiting from the biomimetic structure, the system is simple and effective. Which are extremely snesitive on external factors, especially missing vision signals. In this paper, a virtual view-generation algorithm based on generative adversarial networks (GAN) is proposed to enhance the robustness of binocular vision systems. The proposed model consists of two parts: generative network and discriminator network. To improve the quality of a virtual view, a generative network structure based on 3D convolutional neural networks (3D-CNN) and attentive mechanisms is introduced to extract the time-series features from image sequences. To avoid gradient vanish during training, the dense block structure is utilized to improve the discriminator network. Meanwhile, three kinds of image features, including image edge, depth map and optical flow are extracted to constrain the supervised training of model. The final results on KITTI and Cityscapes datasets demonstrate that our algorithm outperforms conventional methods, and the missing vision signal can be replaced by a generated virtual view.

## 1. Introduction

Composed of left and right cameras, a binocular vision system is a popular form of multi-perspectives computer vision systems [1]. Different from monocular vision system, it can apperceive the spatial information by 2D image data through two cameras, just like a human vision system [2], and it shows a great performance in conducting the localization and mapping of autonomous vehicles. Most binocular systems have protection mechanisms, including anti-seismic design and anti-electromagnetic interference design. These mechanisms ensure the reliability of data transmission at the hardware level. However, it is difficult to deal with the missing data when the image has been blurred due to external interferences, such as if the lens has been polluted by flying insects or mud, or the car body vibration. In this paper, we turn to a virtual view-generation algorithm to enhance the fault tolerance of binocular systems at the software level. It is the task of inferring the different angle of sight based on existing camera vision which plays an important role in image restoration, image enhancement, target recognition and object tracking. In addition, it has been also applied in data augmentation to enhance the performance of models. The difficulty is that the projection from a two-dimensional (2D) plane to three-dimensional (3D) space is an ill-posed problem. Generally, we can find a unique mapping relationship from 3D space onto 2D surfaces according to the projection theory. On the contrary, there is more than one mapping relationship between 2D plane and 3D space due to the lack of depth information. So it is hard to determine a proper one.

Many researches focus on reconstructing a 3D model, because when the geometric models have been reconstructed, the 2D image can be obtained easily by projection theory. This is called a model-based rendering algorithm [3] (MBR). However, the quality of rendering results largely depend on the accuracy of the geometric model and the illumination model. Magnor M. et al. [4] reconstructed 3D static scene geometry, and view-dependent texture maps are generated from all images. Nishino K. et al. [5] proposed a method to sample appearances of a real object with various illumination and viewing conditions, and compress them into the 2D coordinate system which is defined on the 3D model surface generated from a sequence of images. Due to the high cost of model processing and lack of flexibility, it is unsuitable for dynamic scenes.

An image-based rendering algorithm [6] (IBR) generates the virtual view through multi-view images and discards model processing completely. Fehn C. et al. [7] proposed a depth image-based rendering algorithm (DIBR) which is used to generate a virtual view by warping one color image and depth-image in 3D television (3DTV) systems. Sharma M. [8] supported virtual pan-tilt-zoom (PTZ) functionalities during 3D view-generation. In order to deal with the missing area of a virtual view-generation algorithm, fast marching method [9] (FMM) is proposed. It repairs the boundary of holes firstly, and then steps into the centres of holes. Although FMM is capable to handle the missing area, the regeneration processing cost much more time in the dynamic scene. Ce Zhu et al. [10] tried to utilize the occluded information to identify and locate the relevant background pixels around a hole. However, missing area problems and time-consuming problems still exist.

In recent years, deep learning algorithms have achieved great progresses in many image processing fields [11,12], e.g., image segmentation, object detection, super-resolution. John F. et al. [13] is the pioneer of deep learning in virtual view-generation. They take five images as input data to train a view synthesis model which contains two sub networks. One is the selection tower network which is applied to produce a depth probability map, and the other is color tower network producing the best color for each depth. Tingh Zhou et al. [14] presented a framework that re-parametrizes image synthesis to predict the appearance flow field between the inputs and the outputs. However, both of those two methods need much time to process one frame.

In order to enhance the safety of a vehicle and improve the reliability of binocular vision system in the self-driving scene, a virtual view-generation algorithm based on GAN is proposed in this paper. It improves the IBR method by simplifying the generation processing and reducing the dependence on the camera matrices. The main contributions of this work are as follow:

1. A novel GAN structure based on 3D-Dense-Attentive (3DDA) structure is proposed to extract time-series features and to generate the missing area.
2. A composite global loss function based on L1 and SSIM is proposed to improve the overall quality of reconstruction results.
3. The attentive network is designed to obtain a local mask from a depth map and edge map. Then, the mask is used to locate the essential detail which the model should consider.

The outline of the paper is presented as follows: Section 2 introduces feature extraction methods which are applied in preprocessing. Section 3 is our main work. Section 4 shows the experimental results and discussion of models, which are tested, respectively, on two self-driving datasets KITTI [15] and Cityscapes [16], and the results are estimated by the general image quality assessment criteria. Finally, combined with the ORB-SLAM2 system [17], the virtual view can be used to trace object trajectory successfully. In Section 5, a conclusion is given.

## 2. Feature Extraction

The raw data will introduce much noise during training. To reduce the negative effect, three image features, optical flow, depth map and edge map, are extracted to represent key information. The extraction methods are shown as follows.

### 2.1. Optical Flow

**Figure 1:**
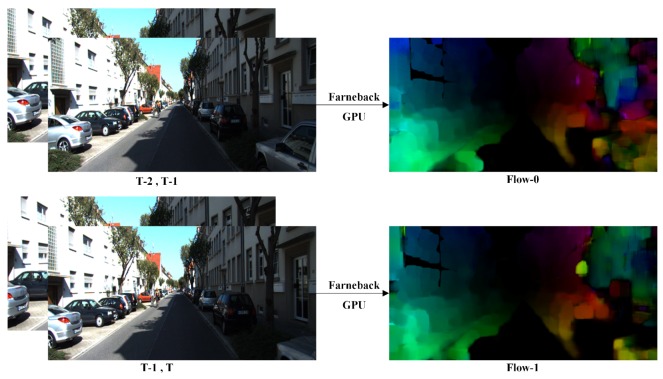
Optical flow from Farneback algorithm.

Although the sparse optical flow [18] is mainly used in the object tracking task, it is too sparse to complete the reconstruction mission at pixel level. Therefore, dense optical flow is introduced to replace the sparse optical flow. With the Farneback method [19], the dense optical flow feature is extracted within two continuous frames. With the GPU acceleration of CUDA operation, the processing speed is raised to 25 frames per second. Thus the Farneback method could meet real-time requirements, and the result is shown in Figure 1.

### 2.2. Depth Map

**Figure 2:**
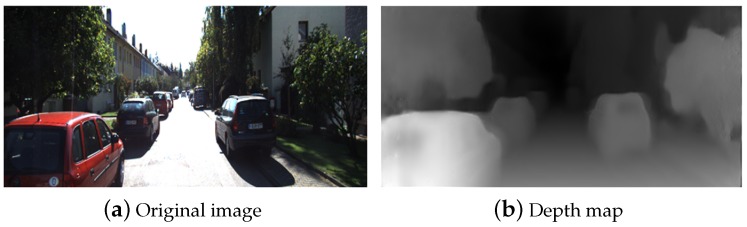
Depth map from mono-depth method.

Depth map indicates the distance information between the object and the camera. There are many methods to get depth maps. In a dynamic environment, a depth camera can provide an accurate results, but the range of depth camera is narrowed. The binocular vision system can obtain the depth map by left and right disparity [20]. The disadvantage is obvious that it requires binocular calibration and lacks redundancy. Recently, depth map estimated by monocular vision has shown great potential to overcome the disadvantage of the above methods. In order to solve the lacking of the ground truth depth map, an unsupervised monocular depth estimation model has been proposed by Godard [21]. Constrained by the left–right disparity consistency Equation (1), an approximate depth map is obtained.

(1) $$C_{lr}^{l}=\frac{1}{N}\sum_{i,j}\left|d_{ij}^{l}-d_{ij+d_{ij}^{l}}^{r}\right|$$

where $d_{ij}^{l}$ is the estimated disparity value of left camera and $d_{ij+d_{ij}^{l}}^{r}$ is the estimated disparity value of left camera based on the right estimated disparity. Although the estimated results are not equal to the ground truth, it is still regarded as one kind of useful features to reconstruct virtual view without RGB-D camera. The depth map is shown in Figure 2.

### 2.3. Edge Map

**Figure 3:**
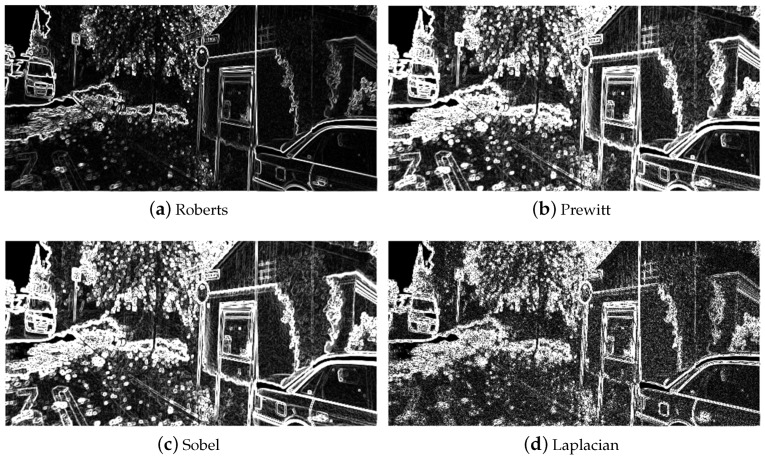
Image edge map with four operators.

Edges are the junction of different attribute regions in the same image. These locations are always ambiguous in image restoration task. According to computer graphics, there exists discontinuity on the edge of an object in gray scale images, and image gradient can be regarded as a source of the edge information. Therefore, an edge map can be obtained by edge extraction of the original image. The extraction steps are shown in Equations (2)–(4):

(2) $$\nabla f=\left[\begin{array}{c} g_{x}\\ g_{y} \end{array}\right]=\left[\begin{array}{c} \frac{\partial f}{\partial x}\\ \frac{\partial f}{\partial y} \end{array}\right]$$

(3) $$\theta =\arctan\left[\frac{g_{y}}{g_{x}}\right]$$

(4) $$M=\sqrt{g_{x}^{2}+g_{y}^{2}}$$

where $g_{x}$ is the gradient on *x* and $g_{x}$ is the gradient on *y*. θ is the direction angle of gradient, and *M* is the magnitude of gradient. Roberts, Prewitt, Sobel [22] and Laplacian operators [23] are the most commonly used methods to extract an image edge. The results are shown in Figure 3.

## 3. Proposed Methods

The generation models based on deep learning has potential in image generation. Varitional auto-encoder algorithm (VAE), proposed by Kingma DP [24], adds constraint on the encoding network and requires the generated latent vector to follow a Gaussian distribution, but the result of VAE is too vague to meet the accuracy requirement of an image. Generative adversarial networks [25] (GAN) proposed by Goodfellow are widely accepted as milestones of deep learning. After that, many approaches based on GAN networks are applied in the image processing. Pathak [26] presented an unsupervised inpainting algorithm to fill the missing areas by introducing an encoder–decoder structure into GAN. The encoder module is used to learn the context and texture details of the raw data, and the decoder module is devoted to predict the missing hole of the image. Isola [27] proposed a pix2pix network based on conditional generative adversarial networks (CGAN). The algorithm learns a relative mapping between different image style pairs. Junyan Zhu et al. [28] presented an approach for learning a general relation map to translate an image in the absence of paired examples. In this paper, the proposed networks are also based on GAN networks, and restricted by global and local loss of functions.

### 3.1. Model Structure

At present, virtual view-generation algorithms use multiple cameras such as an image cue to estimate depth information. However, there exsit joint calibration problems and fault tolerance problems in multi-camera schemes. With the assumption that most objects we encounter every second do not vary drastically in shape, we can make it possible to infer virtual view from a single image. In the field of self-driving, the perceptual error caused by the dynamic scene brings great challenges to vision perception. Different kinds of intrinsic and extrinsic matrices further influence the image processing.

**Figure 4:**
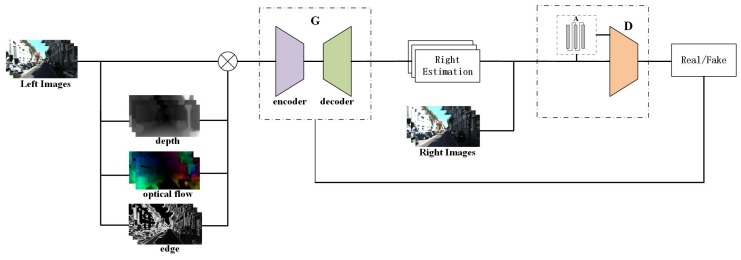
3DDA-GAN networks composed by three sub-networks. G is generative network, D is discriminator network, A is attentive network.

The capability of virtual view-generation based on GAN is improved by a novel structure which can extract the time-series features properly, which is shown in Figure 4. The structure of generative model, discriminator model and attentive model are shown as follows:

#### 3.1.1. Generative Network

The generative network is designed to generate data which should be consistent with the ground truth. Compared with the DIBR algorithm, the generative network can avoid the problem of image distortion caused by change of camera parameters. Moreover, data generation is faster since it has no iterative step of FMM operation.

However, there are two problems in the virtual view-generation task. One is how to learn a translation relationship between different viewpoint angles, and the other is how to fill the missing area. Integrating image style transfer learning and image segmentation algorithms [28] into the generative model is supposed to be an ideal solution for the first problem under the static situation. The generative model shall pay more attention to the essential position of the target due to the image segmentation, but under the driving scene, taking the motion evaluation of vehicle states into image reconstruction makes this problem more complicated [29].

**Figure 5:**
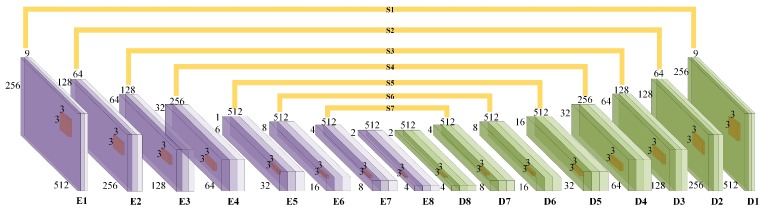
Generative Network.

Although a 2D-CNN [30] network can deal with the second problem under the static scene, the quality of generated virtual view is always unsatisfactory. To overcome this defect, time-series information is used by our method. As shown in Figure 4, three successive frames are concatenated to a cube as input data according to channel axis. Instead of the 2D kernels, the 3D kernels extract the time-series features from the cube. This idea is inspired from 3D-CNN [31] which introduces a network for human action recognition. A 2D convolution layer of U-net is replaced by a 3D convolution layer with convolution filters of 3 × 3 each [32]. In Figure 5, the model contains eight encoder layers and eight decoder layers which are connected by eight skip layers. Combined with high level features through skip connection, the decoder layer maintains the variety of features. This operation neither increases the number of network parameters nor computational complexity, but the performance of a generative model is improved. To the best of our knowledge, this structure is first applied in the virtual view-generation problem.

Compared with DIBR, the generative model integrates the learning of mapping relationships and inpainting processing, greatly shortening the running time. Due to the 3D-Unet structure, the model can regenerate three continuous frames simultaneously and reduce the sampling frequency. After training, the model can be transplanted into any binocular vision system.

#### 3.1.2. Discriminator Network

The discriminator is another essential part of GAN networks. It will be trained firstly to rectify hyper parameters of the generative network forward to the targets. In GAN networks, the discriminator model changes the adversarial problem to a supervised binary classification problem. It produces a true or false signal about similarity statistics between the input data and target data, and the structure of discriminator is a simple multi-layer neural network. If the discriminator has a deeper and more complex structure like the Residual Networks (ResNet) shown in Equation (5), the regression capacity will be improved [33], but back propagation of the gradient will be prevented when the discriminator sends back the same signal. The structure of the discriminator shall be designed more exquisitely so that a generative model can be obtained.

(5) $$x_{i}=H_{i}\left(x_{i}\right)+x_{i-1}$$

(6) $$x_{i}=H_{i}\left(\left[x_{0},x_{1},\ldots ,x_{i-1}\right]\right)$$

where *H* operation represents the cascading of all the previous layers outputs (0,1,…,i−1).

**Figure 6:**
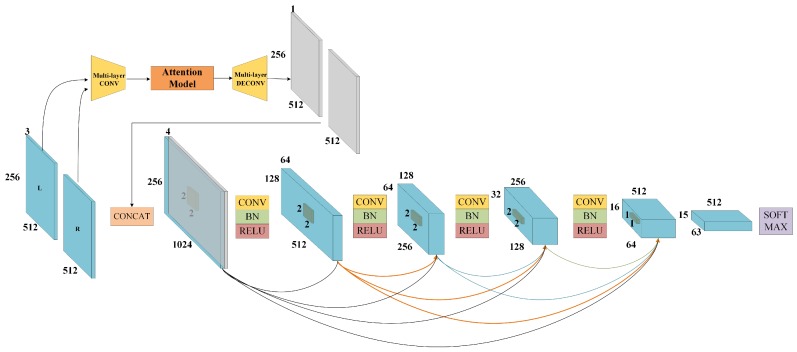
Discriminator Network.

The discriminator is composed of two sub networks, an attentive network and a dense network, which are shown in Figure 6. The former network produces attentive matrix. The architecture will be introduced in the next section. A conventional CNN structure has been used to extract features and get the probability of image similarity. However, the discriminator lacks effective discriminatory ability with simple structure. Inspired by Dense Networks [34] (DenseNet), the Dense Block is introduced into the network. The idea of DenseNet is to shorten the connection between the front and the next layers, as shown in Equation (6), which is first proposed by Huang and mainly refers to ResNet, which has a deeper structure and better classification ability, and this function will maintain the variety feature maps during the convolution operation. Therefore, a dense block structure has several advantages. It solves the problem of gradient vanish and improves feature maps propagation in networks.

The CNN structure of a discriminator network is improved by Dense Block as shown in Figure 6, which is composed of five convolution layers and four dense blocks. In the early stage of training, the depth of discriminator model will be limited, because gradient vanish frequently occurs in the discriminator network with a deeper structure, and the dense block structure is helpful to make features more available in each layer.

#### 3.1.3. Attentive Network

**Figure 7:**
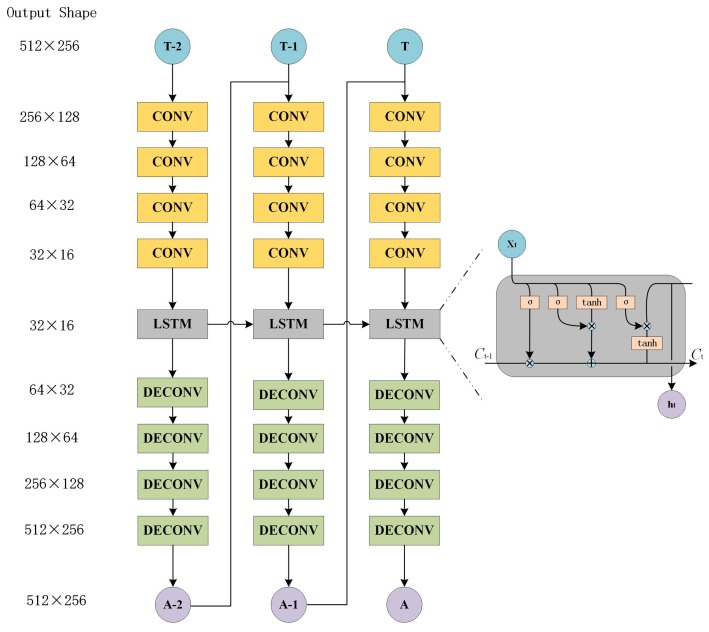
Attentive Network.

When most human beings are observing an object, the first step is to obtain the profile of the object. Then, eyes will take in more attention regarding the detail of targets, such as shadow, color, and texture. This processing is called an attentive mechanism [35]. For improving recognition of local details, the attentive model is introduced into a generative network to produce an attentive matrix (AM). This matrix represents a weight of each pixel and it can be used to extract the essential details simulating the human vision behavior. The attentive model is composed of recurrent neural network and auto-encoder structure, shown in Figure 7, whose weights are shared with the attentive model of discriminator networks.

The attentive network is composed of three standard LSTM layers. LSTM is an extensive form of RNN [36], first proposed by Juergen Schmidhuber [37]. Compared with the results of ordinary neural networks, the results of each hidden layer of RNN are related to the current input and the last hidden layer. In this way, the results of RNN have the characteristics of several previous memories. The LSTM operation can be described as follows in Equations (7)–(12):

(7) $$f_{t}=\sigma \left(W_{f}\left[h_{t-1},x_{t}\right]+b_{f}\right)$$

(8) $$i_{t}=\sigma \left(W_{i}\left[h_{t-1},x_{t}\right]+b_{i}\right)$$

(9) $${\tilde{C}}_{t}=\tanh\left(W_{C}\left[h_{t-1},x_{t}\right]+b_{C}\right)$$

(10) $$C_{t}=f_{t}C_{t-1}+i_{t}{\tilde{C}}_{t}$$

(11) $$o_{t}=\sigma \left(W_{o}\left[h_{t-1},x_{t}\right]+b_{o}\right)$$

(12) $$h_{t}=o_{t}\tanh\left(C_{t}\right)$$

where $x_{t}$ is the input of current cell, $h_{t-1}$ is the output of previous cell, σ is sigmoid function. $i_{t}$ is the output of input gate layer, which limits information to pour into a cell. ${\tilde{C}}_{t}$ is an alternative vector for updating. $C_{t}$ is updated by state of previous layer and ${\tilde{C}}_{t}$. $o_{t}$ is output of current cell. $h_{t}$ is state of current cell. After four deconvolution operations, AM will be obtained.

### 3.2. Loss Function

Virtual view reconstruction is the goal of the model. In our research, a more sophisticated loss function composed of global loss and local loss is proposed. The global loss leads the network to generate rough information, and the local loss is capable of refining the detail.

#### 3.2.1. Global Loss Function

The pixels represent the properties of object by RGB value and the value scope is (0–255). In conventional methods, those reconstruction loss functions lead the orientation of gradient descent, which are shown in Equations (13) and (14)

(13) $$L\mathrm{os}s_{L_{1}}=\frac{1}{N}\sum_{x,y\in N}\left|I_{t\arg et}\left(x,y\right)-I_{output}\left(x,y\right)\right|$$

(14) $$L\mathrm{os}s_{L_{2}}=\frac{1}{N}\sum_{x,y\in N}{∥I_{t\arg et}\left(x,y\right)-I_{output}\left(x,y\right)∥}^{2}$$

where $L\mathrm{os}s_{L_{1}}$ is the mean absolute error (MAE) and $L\mathrm{os}s_{L_{2}}$ is the mean squared error (MSE). Both of them are common methodss to measure data error of diverse data sources in text analysis, image processing and stocking prediction. However, MSE has a poor performance in the image processing field, especially in image similarity evaluation. Image data is composed of pixel matrix, MSE will ignore the association attributes such as object structure, luminance space, and colour continuity. If there are two images with the same objects but generated under different lighting conditions, MSE gives a lower similarity score, which is supposed to show a high score. Peak signal-to-noise ratio (PSNR) outperforms MSE, but still has defects leading to contradictions with human visual perception [38].

With the development of image processing, the structural similarity (SSIM) index [39] and feature similarity (FSIM) index [40] have been proposed to estimate the image quality. SSIM and FSIM are based on the image degradation model, and could extract structural information instead of calculating absolute pixel error. A number of experiments show that SSIM and FSIM have better performance compared to PSNR and MSE. Here, SSIM is chosen as the loss function component of our generative network, mainly because it is differentiable and more efficient. SSIM contains three comparison measurements, luminance, contrast, and structure, as shown in Equations (15)–(19).

(15) $$SSIM\left(x,y\right)={\left[l\left(x,y\right)\right]}^{\alpha }\cdot {\left[c\left(x,y\right)\right]}^{\beta }\cdot {\left[s\left(x,y\right)\right]}^{\gamma }$$

(16) $$l\left(x,y\right)=\frac{2\mu_{x}\mu_{y}+c_{1}}{\mu_{x}^{2}+\mu_{y}^{2}+c_{1}}$$

(17) $$c\left(x,y\right)=\frac{2\sigma_{x}\sigma_{y}+c_{2}}{\sigma_{x}^{2}+\sigma_{y}^{2}+c_{2}}$$

(18) $$s\left(x,y\right)=\frac{\sigma_{xy}+c_{3}}{\sigma_{x}\sigma_{y}+c_{3}}$$

where l(x,y) is luminance, c(x,y) is contrast and s(x,y) is structure. According to Ref. [38], the α,β,γ are generally set to 1 and $c_{3}=c_{2}/2$, the specific form of SSIM as follow:

(19) $$SSIM\left(x,y\right)=\frac{\left(2\mu_{x}\mu_{y}+c_{1}\right)\left(2\sigma_{xy}+c_{2}\right)}{\left(\mu_{x}^{2}+\mu_{y}^{2}+c_{1}\right)\left(\sigma_{x}^{2}+\sigma_{y}^{2}+c_{2}\right)}$$

SSIM is blunt to brightness and color, but it shows better performance in retaining high frequency information, and $L_{1}$ can keep color brightness characteristics better. The global loss function is shown in Equations (20) and (21):

(20) $$Loss_{SSIM}=1-SSIM\left(x,y\right)$$

(21) $$Loss_{global}=\phi Loss_{L1}+\left(1-\phi \right)Loss_{SSIM}$$

In Table 1, we found that when ϕ=0.4, the MSE and PSNR are the best. In the SSIM field, the result of ϕ=0.4 is very close to ϕ=0.8. Besides, when we take $L_{2}$ loss function instead of $L_{1}$, the quality of reconstruction decreases obviously. The reason is that $L_{2}$ can inhibit large errors, but tolerates small errors. Even though $L_{2}$ can handle Gaussian noise, there are various types of noise in the reality scene which prevent it from achieving ideal performance. Consequently, $L_{1}$ loss function and ϕ=0.4 are chosen to be applied in global loss.

#### 3.2.2. Local Loss Function

The global loss function concentrates on overall evaluation of reconstruction error and ignores the details of images. To improve the quality of virtual view, a local loss function is proposed to refine this problem. The biggest challenge is how to locate the local mask.

Firstly, the location mask can be obtained according to the edge map which is calculated by the image gradient method and attention model. Each value of the attentive matrix is the probability of the corresponding pixel. When the object is close to the camera, the reconstruction error becomes bigger according to the perspective theory. So the attentive matrix is still filtered by $T_{m}$ for focusing more attention on the edge areas, which is shown in Equation (22):

(22) $$I_{img\_mask}\left(x,y\right)=\left\{\begin{array}{cc} 1 & AM\left(x,y\right)\geq T_{m}\\ 0 & others \end{array}\right.$$

The smaller the $T_{m}$ is, the greater the receptive field of $I_{img\_mask}$. It brings about more noise into local loss. On the contrary, when $T_{m}$ is much larger, the receptive field becomes small and the edge mask too sparse to make the local loss function converge.

**Figure 8:**
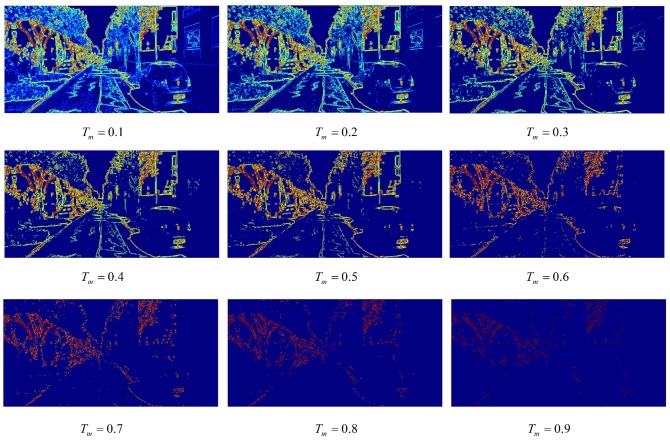
Edge attentive heat map with different $T_{m}$.

The attention heat map of an edge can been seen in Figure 8. The blue area represents the low attentive probability and the red area denotes the high attentive probability. $T_{m}=0.6$ is chosen for Equation (22), because edge attentive matrix can represent a key information mask and decreases the influence of noise.

Secondly, the depth map provides another location mask. We know that the depth map contains semantic information. If there is only a tiny difference between two depth sets in the same map, those two sets are considered to have the same distance. When those three time-series depth maps are used as inputs, the depth attentive matrix can be calculated by an attention model. It also needss to be filtered by $T_{d}$, as shown in Equation (23):

(23) $$I_{depth\_mask}\left(x,y\right)=\left\{\begin{array}{cc} 1 & AM\left(x,y\right)\geq T_{d}\\ 0 & others \end{array}\right.$$

**Figure 9:**
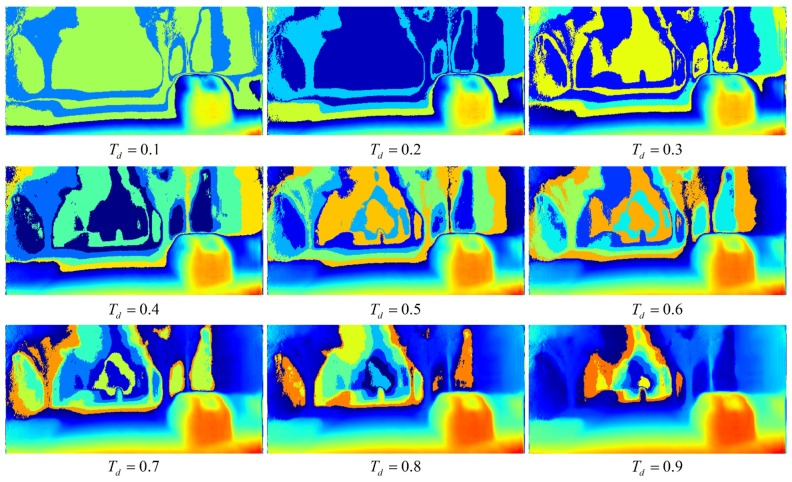
Depth attentive heat map with different $T_{d}$.

Because the low probability mask will mislead the attention onto an unimportant area, it demands more attention to near-sight obstacles, as Figure 9 shows. The meaning of color is the same as in edge attentive heat map. When the value of $T_{d}$ is lower, a higher attention area will be filtered. However, the attention area will focus on the far-sight obstacles when the $T_{d}$ close to 1. $T_{d}=0.7$ is chosen for Equation (23), because a depth attentive matrix focuses on near-sight obstacles. Then, two types of local masks are added together in Equation (24):

(24) $$I_{mask}\left(x,y\right)=I_{img\_mask}\left(x,y\right)⨀I_{depth\_mask}\left(x,y\right)$$

where the operator ⨀ represents the element-wise OR-gate operation.

Thirdly, the loss function is designed to focus on the detail of a foreground scene. Generally, far-sighted objects have relatively small values of optical flow, while near-sighted objects have relatively large values of optical flow, especially moving objects in dynamic scenes. Thus, when optical flow is is applied in judging dynamic objects, with a bigger difference is, the speed changes rapidly. Finally, the local mask can be obtained by two successive optical flows as shown in Equation (25), and the operator × is defined as the element-wise product.

(25) $$I_{mask\_local}\left(x,y\right)=I_{mask}\left(x,y\right)\times Norm(I_{optical\_flow})$$

(26) $$\begin{array}{c} Loss_{local}=\frac{1}{N}\sum_{x,y\in N}\left\{|I_{t}\left(x,y\right)-I_{p}\left(x,y\right)|\times I_{mask\_local}\left(x,y\right)\right\} \end{array}$$

Combined with an image edge mask, a depth map mask and the optical flow, $Loss_{local}$ are shown in Equation (26). The local loss can be extracted and applied in the quality measurement when $T_{m}=0.6$ and $T_{d}=0.7$.

## 4. Experiment and Discussion

### 4.1. Benchmark

To verify the capability of the proposed method, two public self-driving datasets are introduced in this section. The KITTI [15] dataset is an autonomous driving scene dataset and contains the plenty of data from multi-sensors in the autonomous driving scene, such as a binocular vision system, lidar, and GPS. The odometry dataset of KITTI is used to train and test the proposed algorithm, and there are 21 outdoor scenes of stereo cameras with images of resolution of about 375 × 1241, and the sampling frequency is 10 Hz; 15,000 left images and 15,000 right images are processed to 5000 image pairs for training and 6000 images for testing. All images have been resized to 256 × 512. The Cityscapes [16] dataset contains plenty of street scenes in 23 different cities. It collects the data from a stereo vision system in daytime with good weather conditions in spring, summer, and fall. The resolution of images is 1024 × 2048 and the sampling frequency is 17 frames in one second. The data preprocessing is as the same as the KITTI dataset, and all images are down-sampled to 256 × 512. We perform data augmentation process just following [21].

### 4.2. Network Structure

In this section, our algorithm is compared with other models to verify its effectiveness.Those five models are listed in Table 2.

The first model, DIBR, is a conventional algorithm to restore the missing signal without training. The latter four models are trained on the KITTI dataset and Cityscapes dataset separately. The baseline is a pix2pix model [27] which is based on CGAN. It directly inputs raw data instead of random noise into the generating network and achieves better performance. Model A improves the baseline model by global loss. Model B introduces 3D convolution layers to deal with the time-series data. The dense block structure is introduced to improve the discriminator network, and the attentive model is applied in mask generation. Therefore, model B is called 3DDA-GAN. The local loss is utilized in model C. In this section, a Sobel operator is used to extract image gradient features, and 5000 pairs of left–right images are taken to train our models and 1000 pairs of images to test them. The image evaluation indices refer to the previous section. K represents the KITTI dataset, C denotes the Cityscapes dataset.

Table 3 shows that model A, B, and C all outperform DIBR and the baseline according to MSE, PSNR, and SSIM in two datasets. Comprehensively comparing baseline with three modified models, it is obvious that introducing global loss to the baseline (model A) helps improve its performance, utilizing 3DDA-GAN (model B) and further improving the accuracy of the model, and taking local loss into consideration allows model C to obtain the best performance.

**Figure 10:**
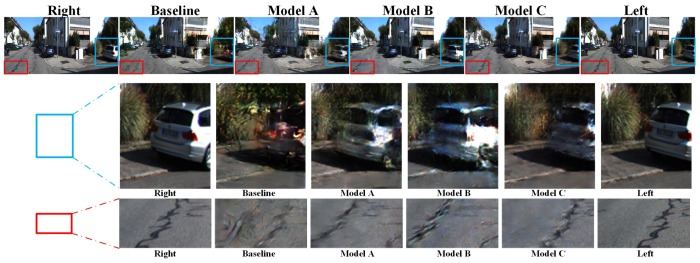
Compared with detail of left vision reconstruction, the blue box tfocuses on the right area and the red box on the left area.

With training processing, we obtain a model learning the mapping function between left and right features. When the left image is missing, the right image can be used as input in virtual view-generation. The comparison result is shown in Figure 10. According to the theory of binocular vision, the different regions always occur in the left-side or right-side. Thus, two regions of every image are selected for comparison in this experiment. The blue box focuses on the right area and the red box focuses on the left area. Although the left virtual view reconstruction of model C is not the same as the ground truth, it approaches the ground truth and shows the best performance in comparison of texture regeneration and left–right consistency with other models.

Our virtual view-generation algorithm is applied in automatic driving systems. Therefore, efficiency is an essential measurement of the algorithm. According to practical requirements, the reconstruction time shall be shorter than the sampling period. The sampling period of the KITTI dataset is 1/10 s, and that of Cityscapes is 1/17 s. In this experiment, there are two indexes measuring inference time of five models. PRT represents the inference time (in seconds) of the reconstruction process for each model, and FRT denotes the inference time (in seconds) needed for every frame reconstruction process. It should be emphasized that, with model B and C, there are three frames which are regenerated in each process. The others generate one frame.

**Figure 11:**
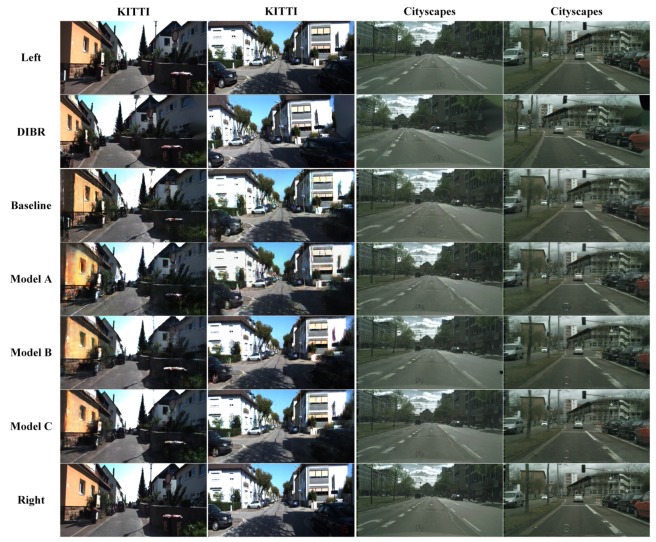
Right vision reconstruction. Left image is the input. Right image is the ground truth. In the experiment, the outputs of five methods are compared in KITTI dataset and Cityscapes dataset.

According to Table 4, the FRT of models B and C are all smaller in terms of the sampling period. Therefore, our model meets the real-time requirement, which mainly benefits from the 3DDA-GAN structure. There displays a more intuitive contrast in Figure 11. Considering the quality and inference time of image regeneration, we believe that although the new model has a complex structure, its performance is much better than that of traditional algorithms and baselines.

### 4.3. Multi-Feature Selection

The Sobel operator is utilized to extract image edge information in our algorithm. To verify the reasonability of choosing a Sobel operator, an addition experiment is conducted by comparing Sobel with other three commonly used operators, namely Roberts, Prewitt and Laplacian, and the test model is the baseline model.

In Table 5, the results show that the Sobel operator outperforms other operators upon all above-mentioned image evaluation indices on the Cityscapes dataset. Although it does not outperform other operators on the KITTI dataset, it still ranks the second on MSE, the third on PSNR, and the second on SSIM. In addition, the Sobel operator has a simple structure. By comprehensive comparison, Sobel operator is considered an appropriate choice.

**Figure 12:**
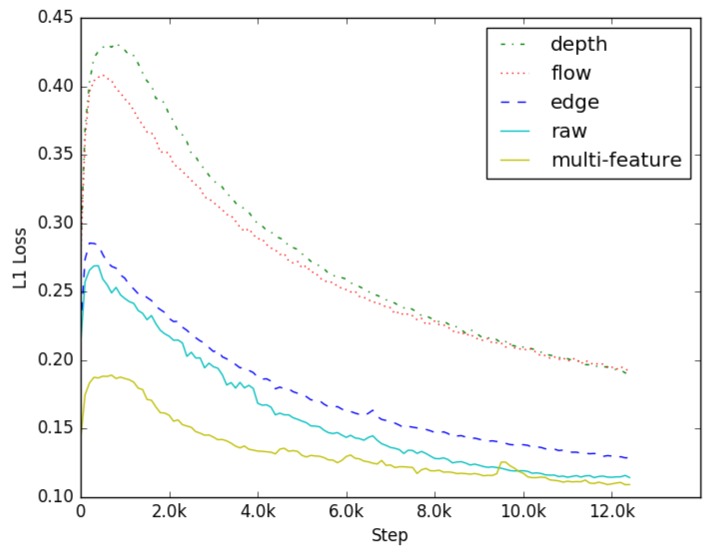
Comparison of L1 with different inputs.

The inputs of conventional GAN networks are those of random noise. With training, the result of generative network can theoretically approach the real data. However, there are not plenty of constraints enhancing the generative ability of the model. For larger size images, random noise is not a proper input. CGAN is an extension of GAN. Additional information is added into both generator and discriminator as a condition, which makes up for the weaker ability of generation. To further improve the quality of generation and accelerate the training speed, a multi-feature fusion method is proposed in this paper, which inputs raw data with the image shape features and spatial features as additional information in the generation model. The shape feature of the image is edge information, and the spatial feature is depth information and optical flow information. It makes the model learn prior data distribution in training, and the results with different feature information are compared in Figure 12.

The result shows that the shape feature is much better than the spatial feature when using a single type of feature to restore the virtual view, but they all have poorer quality of images and slower convergence rate than raw data. Using multi-feature fusion in training, we find that the initial value of loss is much lower and the model can converge more quickly. The reason is that the model has learnt the prior data distribution according to the multi-feature early in the training. However, the more information available as input, the longer inference time the model has. To reduce the inference time of a generative network, edge map, depth map, and optical flow are selected as additional information.

### 4.4. Blurred Image Regeneration

**Figure 13:**
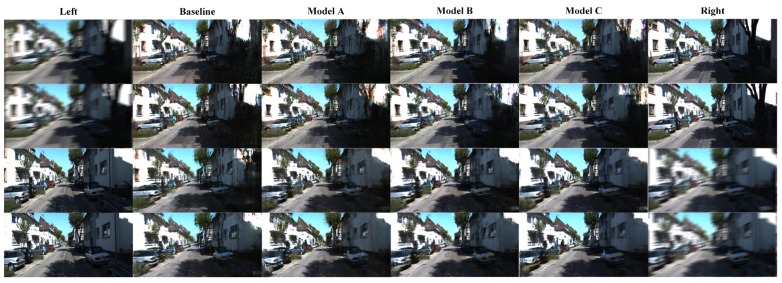
Virtual view reconstruction based on blurred images. The top two rows are based on blurred left image sand the bottom two rows are based on blurred right images.

During driving, the body of a car inevitably vibrates, which will cause blurred data collected by the camera. To validate the regeneration ability of the 3DDA-GAN model based on blurred images, the training dataset and testing dataset are redesigned. We use a motion blur operator to process one of the views. That operation makes the task of virtual view regeneration more difficult. The performance of 3DDA-GAN model in blurred image regeneration task is shown in Figure 13.

According to Table 6, the MSE statistics show that the blurred images indeed influence the quality of virtual view, but the proposed model still has the best performance in quantitative comparisons. Moreover, it approaches the result when the input is not blurred. So, the 3DDA-GAN model is robust in unsteady environment.

### 4.5. Polluted Image Regeneration

Besides the blurred data, the lens of the visual sensor is often polluted by flying insects or mud, leading to the problem of missing data. In this condition, conventional algorithms are no longer competent to generate a virtual view. To verify the model-generation capability in this circumstance, the training and testing datasets are reprocessed by polluted operation. Due to the location of the lens contamination being relatively fixed, a 40 × 40 block of pixels is set to zero in one of the perspectives to simulate the flying insects and mud.

**Figure 14:**
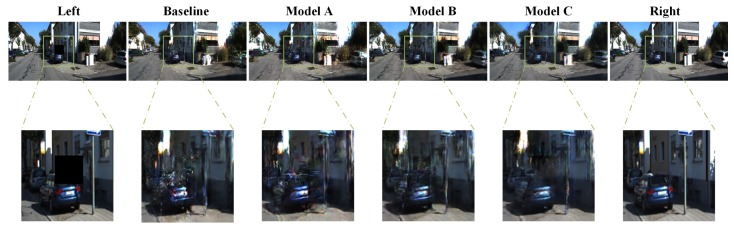
Virtual view reconstruction based on polluted images. The black area in the left image simulates flying insects or mud. The bottom row shows the detail of regeneration.

As shown in Figure 14, the black square in the left image is used for simulating flying insects or mud. Based on this polluted left image, regenerating the right virtual view is a great challenge to algorithms. Comparing these missing areas of right virtual view reconstructions in Table 7, model C shows the best performance.

### 4.6. ORB-SLAM

ORB-SLAM2 was proposed by Taylor Guo, which has better performance over ORB-SLAM [41], especially in binocular cameras and RGB-D cameras. It introduced a visual SLAM technology in real-time robotic route planning and map reconstruction, which contains four parts: tracking, local mapping, loop closing, and local bundle adjustment. This method needs left and right camera images as inputs and extracts several key points using an ORB feature detector with rotation invariance.

**Figure 15:**
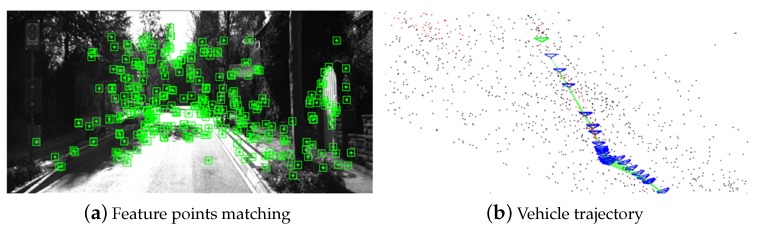
3DDA-GAN applied in ORB-SLAM2.

There are two outputs of ORB-SALM2. The one is feature points matching on left and right vision images. The green points in Figure 15a are key points which are matched by ORB features. The other is an estimation of vehicle trajectory in world coordinate, which is shown in Figure 15b. In this experiment, left images and restored by baseline, model A, model B, and model C are fed into the ORB-SLAM2 system to estimate trajectory. To estimate the accuracy of generated virtual view, the relative error rate is introduced, which measures the difference between estimation trajectory and the ground truth. Sequence 11 of the KITTI odometry dataset is used as test data.

**Figure 16:**
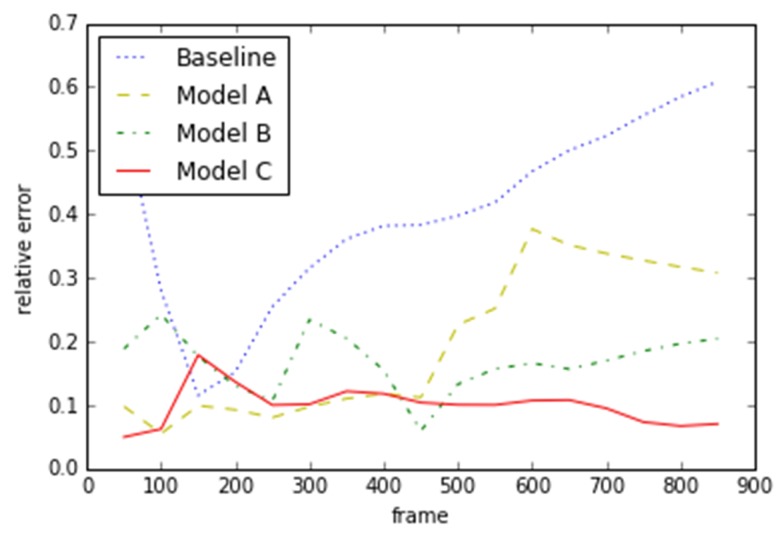
Trajectory relative error of Sequence 11.

As Figure 16 shows, the *x*-axis represents frame number and the *y*-axis corresponds to the relative errors of different models. The baseline model (blue line) has the highest relative error. With the modification of model A, model B, and model C, the results improve continuously. Model C (red line) outperforms the others in the KITTI dataset. According to the statistics of trajectory relative error, as shown in the Table 8, model C is optimal in all the indicators. That means this algorithm has stable performance.

ORB-SLAM2 also supports the monocular mode. When one vision of stereo system is corrupted, monocular mode and binocular mode are two choices. These two ways are compared in the following experiment, respectively. In monocular mode, real left data points (as inputs) are fed into the system, and in the binocular mode, both real left data and generated right data are fed. The test data is still from sequence 11. The right vision data is generated by model A, model B, and model C. In this experiment, $N_{reset}$ means the number of reseta, $N_{traj}$ means the number of tracking frames, $N_{lost}$ means the lost frames, and $R_{traj}$ is calculated by Equation (27). The higher the tracking rate, the better performance the ORB-SLAM2 system has.

(27) $$R_{traj}=\frac{N_{traj}}{N_{traj}+N_{lost}}$$

In the experiment, the reset time measures the reliability of the two methods. When the reset operation occurs, it will cost 3–4 s. As Table 9 shows, the monocular model needs to reset twice during sequence 11. Thus, it is difficult to extract an ORB feature when scenes of continuous frames change a lot. On the contrary, binocular modes have less risks. With the sequence stereo data, the ORB feature points are extracted by a matching algorithm. Although the generated data is not completely equal to the ground truth, the key points are enough to run the SLAM program. According to this result, model B and model C achieve satisfied performance. Finally, model C combined with global loss, local loss, and 3DDA-GAN structure is the best method to generate a virtual view.

### 4.7. Training Details

The 3DDA-GAN network is composed of three sub-networks, generative network, discriminator network, and attentive network. Since the attentive network is one part of a discriminator network, those two networks can be trained synchronously. So we separate training into two steps. The first step is to train a discriminator network, and the second is to train a generative network. Within the alternative training between discriminator network and generative network, the loss function plays an essential role in updating the weights. In Section 3.2, parameter selection improves the capability of loss function. In the global loss function, ϕ is 0.4. In the local loss function, the edge attentive threshold $T_{m}$ is 0.6 and the depth attentive threshold $T_{d}$ is 0.7. During training, we use a mini-batch size of eight to accelerate the convergence, and the optimization operation follows the training of CGAN [26]. In terms of the discriminator optimization, we select an Adam solver with an initialization learning rate of 0.0002 and a momentum term of 0.5. The optimization of the generative network is similar to the discriminator network. The parameters of the network are updated by a moving average method with a decay factor 0.99.

## 5. Conclusions

It is difficult to improve the redundancy of a binocular visual system, but in this paper, a virtual view-generation algorithm is proposed, which can rapidly restore the corrupted vision based on another vision signal. The model contains three key improvements which are local loss, global loss, and 3DDA-GAN structure, respectively. With this novel structure, our model can learn the spatial information from time-series inputs, and the composite loss function improves the quality of image generation. Moreover, experimental results show that our model achieves the best performance of regeneration quality and inference time using data from the KITTI and Cityscapes datasets. In addition, the proposed model is efficient and robust in blurred view regeneration and polluted view regeneration. Meanwhile, it can also be applied in the classical ORB-SLAM system for tracing trajectory. However, some thresholds of the proposed algorithm are selected experimentally which influences generalization. We aim that in the future, a more robust virtual view-generation algorithm will be studied with adaptive thresholds. In addition, other sensors, like laser radars and millimetre wave radars, will probably improve the visual system, and reinforcement learning will be combined with LSTM to improve the attention mechanism. Finally, safety of autonomous vehicles will be consolidated by virtual view-generation based on 3D-Dense-Attentive GAN networks.

## Figures and Tables

**Table 1:** Parameter of global loss function.

	Loss	MSE	PSNR	SSIM
ϕ=0.1	$L_{1}$	58.96	30.45	0.6485
ϕ=0.2	$L_{1}$	58.88	30.38	0.6382
ϕ=0.3	$L_{1}$	62.56	30.20	0.6388
ϕ=0.4	$L_{1}$	55.67	30.70	0.6631
ϕ=0.4	$L_{2}$	62.55	30.19	0.6419
ϕ=0.5	$L_{1}$	55.84	30.69	0.6623
ϕ=0.6	$L_{1}$	55.77	30.69	0.6687
ϕ=0.7	$L_{1}$	57.46	30.57	0.6558
ϕ=0.8	$L_{1}$	55.75	30.69	0.6787
ϕ=0.9	$L_{1}$	58.66	30.47	0.6614

**Table 2:** Model list.

	Model Structure
DIBR	DIBR + FMM
Baseline	pix2pix
ModelA	pix2pix + $Loss_{global}$
ModelB	3DDA-GAN + $Loss_{global}$
ModelC	3DDA-GAN + $Loss_{global}$ + $Loss_{local}$

**Table 3:** Quantitative comparisons on KITTI and Cityscapes datasets.

	K-MSE	K-PSNR	K-SSIM	C-MSE	C-PSNR	C-SSIM
DIBR	87.08	28.75	0.2778	77.98	29.23	0.3967
Baseline	83.83	28.91	0.3371	46.54	31.55	0.6948
Model-A	66.24	29.94	0.5845	44.49	31.75	0.7401
Model-B	58.96	30.46	0.6317	39.84	32.26	0.7661
Model-C	55.13	30.76	0.6699	39.02	32.35	0.7746

**Table 4:** Inference time of five models (unit: second).

	K-PRT	K-FRT	C-PRT	C-FRT
DIBR	1.219	1.219	1.055	1.055
Baseline	0.0315	0.0315	0.0313	0.0313
Model-A	0.0359	0.0359	0.0442	0.0442
Model-B	0.0791	0.0264	0.0881	0.0294
Model-C	0.0926	0.0309	0.0903	0.0301

**Table 5:** Baseline model with four operators.

	K-MSE	K-PSNR	K-SSIM	C-MSE	C-PSNR	C-SSIM
Roberts	68.87	29.81	0.4865	45.81	31.62	0.7191
Prewitt	69.86	29.78	0.4931	44.68	31.73	0.7308
Sobel	68.84	29.79	0.4923	43.20	31.89	0.7416
Laplacain	68.41	29.82	0.4918	46.58	31.54	0.7046

**Table 6:** Quantitative comparisons on blurred image regeneration.

	K-MSE	K-PSNR	K-SSIM	C-MSE	C-PSNR	C-SSIM
Baseline	85.07	26.87	0.4956	73.33	21.51	0.6424
Model-A	73.43	29.72	0.5538	65.09	23.17	0.6913
Model-B	62.94	30.29	0.5894	62.24	26.34	0.7052
Model-C	60.79	30.31	0.6006	59.47	26.92	0.7102

**Table 7:** Quantitative comparisons on polluted image regeneration.

	K-MSE	K-PSNR	K-SSIM	C-MSE	C-PSNR	C-SSIM
Baseline	68.89	25.04	0.5162	53.79	23.22	0.6972
Model-A	66.49	29.88	0.5856	52.14	24.65	0.7113
Model-B	64.93	30.03	0.6166	46.20	25.34	0.7228
Model-C	55.33	30.72	0.6741	45.88	25.89	0.7247

**Table 8:** Statistics of camera trajectory relative error.

	Max	Min	Mean	Var.
Baseline	60.83%	11.53%	40.30%	0.0201
Model A	37.69%	5.52%	19.77%	0.0129
Model B	24.27%	5.95%	16.87%	0.0019
Model C	17.88%	4.98%	9.97%	0.0008

**Table 9:** The result of monocular mode and binocular mode in ORB-SLAM2.

	$N_{\mathrm{reset}}$	$N_{\mathrm{traj}}$	$N_{\mathrm{lost}}$	$R_{\mathrm{traj}}$
Monocular	2	574	326	63.78%
Model A	1	779	121	86.56%
Model B	0	900	0	100%
Model C	0	900	0	100%
